# Optimizing Farmers’ and Intermediaries’ Practices as Determinants of Food Waste Reduction Across the Supply Chain

**DOI:** 10.3390/foods14132351

**Published:** 2025-07-02

**Authors:** Abdelrahman Ali, Yanwen Tan, Shilong Yang, Chunping Xia, Wenjun Long

**Affiliations:** 1 School of Economics and Management, South China Agricultural University, Guangzhou 510642, China; 20211161015@stu.scau.edu.cn; 2 Agricultural Economics Department, Faculty of Agriculture, Fayoum University, Fayoum 63514, Egypt; 3 College of Economics and Management, Huazhong Agricultural University, Wuhan 430070, China; xcp@mail.hzau.edu.cn; 4 Research Center for Rural Economy in Ministry of Agriculture and Rural Affairs, Beijing 100125, China; 87280931@163.com

**Keywords:** food handling practices, stakeholders’ training needs, horticultural postharvest loss, good agricultural practices, grapes supply chain, shelf life

## Abstract

Improper stakeholder practices are considered a primary driver of food loss. This study aims to investigate the consequences of pre- and post-harvest practices on extending the shelf life of agro-food products, identifying which practices yield the highest marginal returns for quality. Using Fractional Regression Models (FRM) and Ordinary Least Squares (OLS), the research analyzed data from 343 Egyptian grape farmers and intermediaries. Key findings at the farmer level include significant food loss reductions through drip irrigation (13.9%), avoiding maturity-accelerating chemicals (24%), increased farmer-cultivated area (6.1%), early morning harvesting (8.7%), and improved packing (13.7%), but delayed harvesting increased losses (21.6%). For intermediaries, longer distances to market increased losses by 0.15%, while using proper storage, marketing in the formal markets, and using an appropriate transportation mode reduced losses by 65.9%, 13.8%, and 7.9%, respectively. Furthermore, the interaction between these practices significantly reduced the share of losses. The study emphasizes the need for increased public–private partnerships in agro-food logistics and improved knowledge dissemination through agricultural extension services and agri-cooperatives to achieve sustainable food production and consumption. This framework ensures robust, policy-actionable insights into how stakeholders’ behaviors influence postharvest losses (PHL). The findings can inform policymakers and agribusiness managers in designing cost-efficient strategies for reducing PHL and promoting sustainable food systems.

## 1. Introduction

Identifying and addressing the food loss drivers, including improper stakeholder practices, is essential to sustain and maintain the shelf life of agro-food products across the supply chain (SC) for as long as possible [1,2,3]. The Boston Consulting Group (BCG) (Boston Consulting Group https://www.bcg.com/publications/2018/tackling-1.6-billion-ton-food-loss-and-waste-crisis, accessed on 30 April 2025) estimates that by 2030, annual food losses and waste (FLW) (according to the FAO, food loss refers to the share of food lost across the food supply chain before it reaches the final consumer, while food waste represents the share of food wasted by the consumers) will hit 2.1 billion tons, worth USD 1.5 trillion, and decreasing it is crucial to achieve sustainable food system transformation, particularly in developing countries (which import most of their food) [4]. Meanwhile, improper handling and hygiene practices result in food contamination, constantly increasing the percentage of loss across the supply chain [5]. Furthermore, measuring the interaction between socioeconomic factors and stakeholder practices toward reducing the rate of loss across the food supply chain (FSC) plays an essential role in designing an appropriate solution to minimizing the share of lost food at different nodes across the supply chain [6,7]. The marginal benefits of preventing losses should exceed the marginal costs to make the intervention more attractive to relevant stakeholders [8]. Adopting good agricultural practices, including cultivation, harvesting, handling, and postharvest practices, dramatically improves the farmers’ net profit and enables the sustainable use of limited resources in developing countries by reducing the percentage of FLW [9]. Generally, pre-sorting on farms can remove fruits with unacceptable shapes, sizes, and colors during harvesting, grading, and sorting at the farm gate. However, these fruits have the same nutritional value and could be used either fresh or processed to feed humans. Due to their appearance, they are often left in the field [3,10].

The handling practices of agro-food products (such as harvesting, pre-cooling, sorting and grading, packaging, and storing) play a substantial role in preserving quality and extending shelf life [11]. Improper practices or neglecting one of the aforementioned operations lead to quality degradation, especially for perishable and sensitive fruits like grapes. For example, poor harvesting methods, incorrect cooling, and poor storage contribute to the loss of products. Because grapes are very sensitive to temperature, they cannot be kept as a product for long periods. However, the substantial role of agricultural education and extension in disseminating the best farming practices, including postharvest operations for perishable products, is weak in most developing countries [12]. This highlights the urgent need to reform the public agricultural extension institutes to raise small stakeholders’ knowledge, improve their practices and skills, and change their behaviors and attitudes to use limited resources more sustainably [13]. Considering the negative effects of spraying harmful chemicals to promote earlier fruit maturity (color, size, and solidity) or sustain their freshness after harvest, this practice can decrease the fruits’ shelf life and result in food safety problems and negative environmental impacts [14].

Fruits provide essential vitamins and offer high profit margins for small-scale farmers and middlemen. Still, the share of postharvest fruit loss is very high due to a lack of agro-processing facilities and agro-logistic services, especially in developing countries. Varying perishability between different fruit types is a key factor affecting all aspects of this industry; growers must maintain minimum product quality for consumers, which impacts pricing and the percentage of losses [3]. However, farmers and middlemen remain hesitant to adopt the recommended postharvest practices. Efforts to disseminate and encourage stakeholders to adopt these practices can significantly reduce the share of postharvest loss (PHL) in grapes and other fresh fruit and food products (PHL refers to the share of lost or wasted food across the food supply chain, starting from harvesting and extending until the product reaches the final consumer).

Egypt is ranked fourth in the world for table grape production quantity (~1.7 million tons), and growth in this production has significantly increased Egyptian grape exports over the past 15 years [2]. The FAO estimated the share of postharvest loss to be around 18.6%, 5.3%, and 6.7% for the farmers, wholesalers, and retailers in North Egypt, highlighting the critical points of losses, including the harvest, wholesale, and retailer market levels, respectively [2]. Egyptian grape producers and value chain actors are increasingly vulnerable to shifts in global markets due to a lack of marketing information and land fragmentation among small-scale producers. They might delay mature crop harvesting to decrease transportation costs, which significantly increases the loss volume. Small-scale producers lacking export access need new strategies to upgrade the table grape value chain.

The interlink between the grapes supply chain and required post-harvest operations depends on the primary destination market (domestic or export). Furthermore, neglecting these operations (e.g., sorting, packing, grading, etc.) at the farm could lead to an increase in the percentage of food loss at the following downstream stages of FSC [3,15]. Stakeholders’ practices and access to logistics significantly influence the proportion of loss across FSC, and this share varies depending on FSC efficiency, logistics service availability, and stakeholder practices [3,16]. For example, cold transportation and storage services mainly contribute to preserving the product for long periods [2]. Adopting good handling techniques, using appropriate equipment or technology, building capacity, enforcing thorough regulations, and improving marketing can avoid many problems [17]. FLW reduction interventions have garnered more global attention and investment following the global food crisis in 2008, when many countries sought to increase local food self-sufficiency through various strategies, including food loss and waste reduction, particularly in developing countries [2].

Individuals’ preferences are based on the costs, benefits, and risks of the choices in line with their socioeconomic characteristics, so they will only act if the benefit outweighs the costs [18]. In the same way, we assumed (1) the stakeholders will invest to decrease FLW when the value of the saved food outweighs the cost of the intervention (practices or technology) [19]. (2) The interaction between the different proper practices could result in a greater reduction of losses across the supply chain. However, the importance of the correlation between the farmer and middleman characteristics and their practices is emphasized. The previous studies did not analyze the different impacts of the interaction between the stakeholders’ socioeconomic characteristics and their practices toward food loss and waste, which could give an overview of the technical training needed to enhance the capacity of the small farmers and intermediaries to adopt the good agricultural practices, including the best postharvest operations. The current study aims to evaluate and analyze the interaction between stakeholders’ socioeconomic characteristics and their impacts on their practices at the different FSC stages, to determine the appropriate intervention strategies for reducing these losses. The results of this study could help policymakers, agribusiness owners, and international funding donors design sustainable intervention reduction strategies for these losses, achieve a sustainable supply chain, attain environmental preservation, and ensure efficient use of limited resources available.

The recent literature indicates a scarcity of evidence on the impact of stakeholders’ practices as a food loss driver [20]. Furthermore, the majority of earlier FLW research concentrated on calculating the proportion of loss [3,21,22], few studies have investigated the food loss driver, particularly horticultural crops. Furthermore, most of these studies used the macro-secondary data (e.g., food balance sheet, FAO) to estimate the FLW, which neglected the heterogeneity between the stakeholders and the significant role of those small stakeholders in FLW reduction strategies, and they provided only high-level insights about the nature and causes of these losses [23]. Despite the substantial investment in reducing losses, the effectiveness of these interventions remains limited due to a lack of coordination among different FCS actors, the failure to improve infrastructure, and inadequate support for capacity building [24]. Therefore, we aim to measure the interaction between socioeconomic characteristics and stakeholders’ practices, which represents an important step in designing appropriate interventions to minimize this share at different nodes across the FSC.

The novelty of the current work could be highlighted through its theoretical and empirical contribution to a better understanding of the root causes and drivers of FLW at the small stakeholder level. This includes assessing the impacts of socioeconomic characteristics heterogeneity among stakeholders on access to logistics services and its effects on FLW reduction. This study presents evidence-based findings on how socioeconomic characteristics and their interaction with other technological factors influence the percentage of FLW across FSC in developing countries. To analyze the FLW drivers, we started with farmers’ practices during farm operations, then the middlemen handling the product to reach the end users. The following sections include the methodology, data analysis, results, discussion, and conclusion.

## 2. Materials and Methods

### 2.1. Theoretical Framework

The reduction of food loss in agricultural supply chains is fundamentally dependent on the intricate interaction of social and economic behaviors demonstrated by farmers and intermediaries. These participants function within a network of incentives, limitations, and relational dynamics that significantly affect post-harvest losses. Grasping this relationship is essential for creating effective interventions [24]. For example, trust between farmers and intermediaries promotes an exchange of market information, where intermediaries disseminate market standards and demand signals, and farmers communicate production difficulties. This openness fosters improved planning and coordination to synchronize harvests with market requirements and minimize mismatch losses [19]. Furthermore, trust and strong networks lower transaction costs, enable collective investment in loss-reducing infrastructure, improve access to credit and information, and strengthen bargaining power, making economically viable solutions more accessible. Long-term relations encourage collaboration, where the intermediaries might provide credit for agro-inputs that mitigate losses (such as cold storage and transportation) or provide technical support; farmers tend to prioritize working with trusted partners, which may improve quality control.

**Figure 1:**
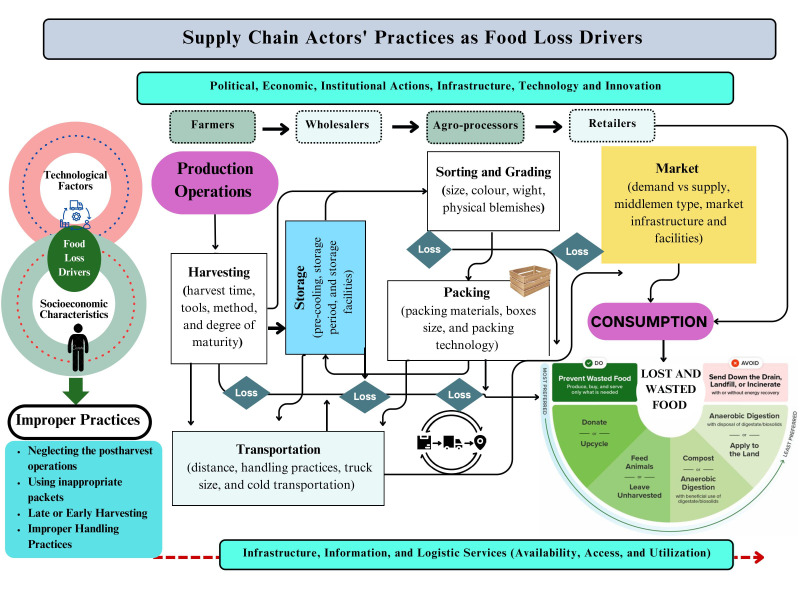
Supply Chain Actors’ Practices as Food Loss Drivers.

The target-measure-act framework has been employed to analyze and measure the impacts of stakeholders’ or individuals’ practices as a food loss driver. This approach has been approved as a recognized approach for developing a rapid result for FLW reduction [25,26]. This study intends to evaluate the interaction between socioeconomic characteristics and the stakeholders’ practices as food loss drivers across FSC to provide a better understanding of how these factors interact. We target the most important actors across FSC who are responsible for the highest share of FLW (farmers and middlemen). Also, the grapes have been considered the main fruit crop in the investigated area. We measured the impacts of these interactions on the stakeholders by employing the rational choice theory, where the stakeholders’ decisions are based on the interaction between the availability, access, and utilization of the facilities and technologies that reduce FLW, and the socioeconomic characteristics of those stakeholders. Finally, the appropriate interventions have been discussed based on the study’s results, as shown in Figure 1.

Rational choice theory (RCT), also known as a rational action or choice theory, is a framework for recognizing and often formally modelling social and economic behaviour [18]. It assumes that individuals’ and agents’ preferences are based on the benefit/cost ratio, where stakeholders will invest in stopping FLW when the value of the saved food outweighs the price of the intervention (practices or technology). Moreover, people use the current resources to maximize rewards, and agents or individuals can incorporate all relevant factors into their preference rankings for the overall alternative. Furthermore, social scientists can indirectly understand agents’ desires through their disclosed decisions. Researchers extrapolate from observable behavior to rebuild the preference hierarchy that governs a rational agent’s decisions.

RCT is used in microeconomics to model human or individual decision-making, where it assists economists in better understanding the behaviour of a society “in terms of individual actions as explained through rationality”. This theory is widely used in economic studies and agricultural policy analysis, which explains human decision-making [27]. Recently, the fundamentals of this theory have been used in many studies to determine the appropriate intervention to reduce food and post-harvest losses [28,29]. The farmer is challenged with a choice of whether or not to adopt post-harvest handling practices to reduce loss and the extent of adoption. So, a farmer faces a tradeoff between incurring additional costs to mitigate loss and risking getting loss. For example, the farmer could risk late harvesting his crop because they wishes the market price might increase or the harvest labor rent might decrease. A rational farmer and middleman will invest in alleviating loss if there is an economic motivation to do so. Additionally, reducing loss increases the amount of the crop available for sale and consumption. Hence, the quantity of the crop saved by reducing loss and the cost of mitigating the losses play a crucial role in the farmer’s decision [29].

Improper practices are mainly a result of a complex interaction across the supply chain between the stakeholders’ socioeconomic characteristics and access to agricultural services. Where the value of crop saved by reducing loss and the cost of mitigating the losses play a crucial role in the farmers’ and intermediaries’ decisions.

### 2.2. Study Area and Sampling Strategy

A multi-stage sampling design has been adopted to select grape farmers and middlemen. According to Yamane, 384 observations are required to justify an infinite population [30]. We used the following equation to determine the sample size.

(1) $$n=\frac{N}{1+N*{\left(e\right)}^{2}}$$

where n is the proposed sample size, N is the total population size, N = (453) for the current study, and e is the level of precision (0.05).

**Figure A1:**
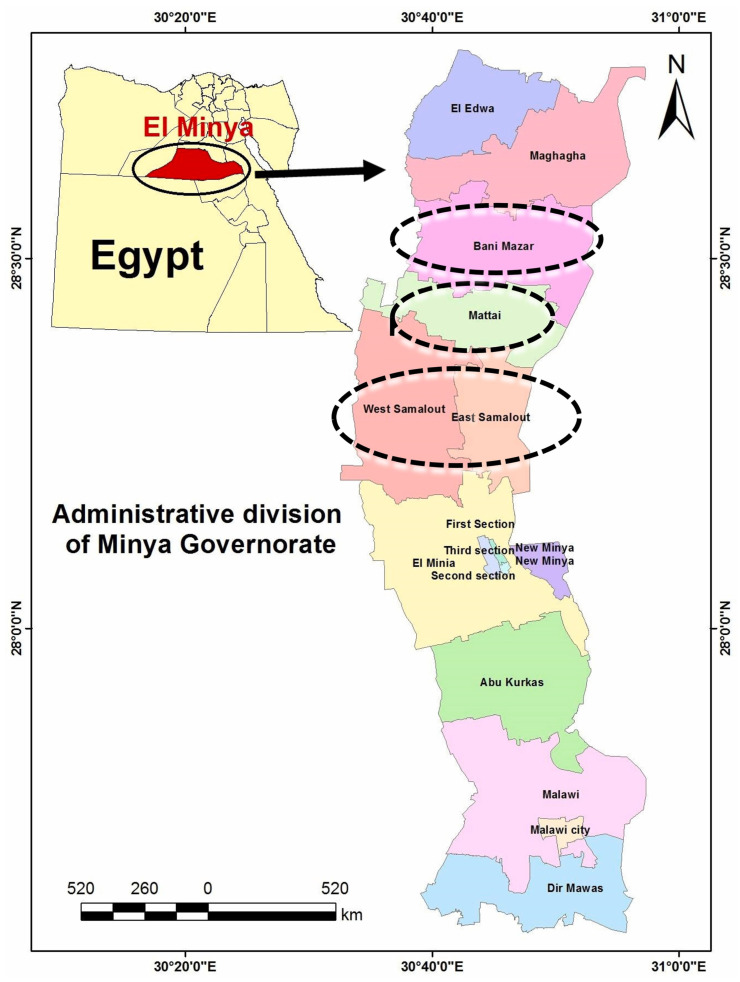
Study area map (*Minya* Governorate, Egypt). The left shows the location on the county map, whereas the right shows the detailed map of the three selected districts in the selected governorate (*Minya*).

In the current study, we only considered small farmers; hence, the proposed sample size would be 213 growers. The sample was chosen from the main grape production governorate in Egypt, according to the cultivated area (*El Minya*, located south of Cairo), as shown in Figure A1. We randomly selected the three main districts (*Bani Mazar*, *Mattai*, *and Samalout*), which represent more than 84% of the total cultivated area in *El Minya* governorate. Then, two villages were selected in each district (based on the grape cultivated area). The respondents’ numbers in each village were determined according to the geometric mean of the number of farmers and the area planted with grapes in each village. Finally, 213 Egyptian grape farmers were randomly selected in these villages. Similarly, we interviewed 130 middlemen involved in the fruit and vegetable trade. We excluded the questionnaires of 13 farmers and 10 middlemen due to incomplete answers.

In this study, we used a structured questionnaire to collect data from the stakeholders through face-to-face interviews during the 2020 season. We developed the two questionnaires (one for farmers and the other for the middlemen). We pretested them, modified them based on the pretest results, and finally, we collected the data. The proposed questionnaires were prepared based on related literature and have been tested through informal group discussions (grape experts and agro-economists) and a pilot study. Table A2 provides more details about the socioeconomic characteristics of the selected farmers and middlemen.

### 2.3. Model Specification

Degradation in quantity and/or quality of agricultural products depends on both farmers’ and middlemen’s practices, which include preharvest and postharvest operations, as follows:

PHL (Y) = F (Pre-harvest practices, Post-harvest practices, Environmental factors, Institutional factors, Interaction effects), which could be presented in the following equation.

(2) $$PHL\_i=G\beta 0+\sum \beta j\;Farmer\_Practices\_ji+\sum \theta k\;Middlemen\_Practices\_ki+\sum \gamma mControls\_mi+\varepsilon_{I}$$

where (PHL_percent) is the dependent or outcome variable, Xi’s predictor or independent variables, including the pre-harvest and postharvest variables, as shown in Table A1. β, γ,θ,∝, δ are coefficients for each predictor and $\varepsilon_{I}\;$ is the error term. We include each practice as a separate regressor to identify which specific practices have an impact. For example, pre-harvest practices are the farm operations such as (irrigation system, pest control (including spraying the chemicals to speed up the maturity or improve the appearance of the final product), timing of harvest, stage of fruit maturity, initial sorting and grading at the farm gate, which the farmers mostly do. While post-harvest practices include (storage conditions, handling, packing, transportation time (cold-chain, distance to the market), etc.), are done by the middlemen, as shown in Table 1. The control variables (Z) include access to information, membership in an agricultural cooperative, farm size, education, etc.

#### 2.3.1. A. Fractional Regression Model (FRM)

Since PHL is a proportional/fractional outcome (e.g., 5%), a fractional regression model is ideal [31]. It uses a logistic or probit link function to ensure predictions stay within [0, 1] (or [0, 100%] after rescaling). Due to the nature of the data, we employed the fractional probit model in the current study.

(3) E(PHL_i∣Xi)=G(β0++∑βj Farmer_Practices_ji+∑θk Middlemen_Practices_ki+∑γmControls_i

G (⋅): Logistic or probit function (e.g., G (z) = $\frac{e^{z}}{{1+e}^{z}}$).

PHL_i = Percentage loss (e.g., 0–100%). The average of percentage losses for each selected stakeholders has been presented in Table A2.

#### 2.3.2. B. Addressing Interaction Effects:

We evaluated the interaction effects to capture synergies or trade-offs between stakeholders’ practices, where interactions between the different practices might influence the percentage of PHL.

(4) E(PHL_i∣Xi)=G(β0+β1 Practice_i+θ1 Practice_k+δ(Practice_i∗Practice_k)i+γControls_i

A negative δ implies that combined practices reduce losses more than their individual effects.

For robustness checks, we compared the results of FRM with Ordinary Least Squares (OLS) {with PHL logit-transformed; $ln(\frac{PHL}{100-PHL}$)}.

The variables were investigated to determine whether they affected the PHL. Then, the factors with an effect were used in the model to determine whether they had a negative or positive effect on the PHL. At that time, some of the affected variables were examined as dummy variables, assuming that the stakeholders adopting and applying the best agricultural practices are (1) and otherwise (0). We checked the autocorrelation and multivariate collinearity, revealing no extreme multivariate collinearity in the data.

## 3. Results and Discussion

Adopting the best agricultural and handling practices will extend the shelf life of agrofood products and consequently decrease the percentage of loss, highlighting the importance of agricultural extension in disseminating information among farmers and other actors across FSC [32]. The following section presents and discusses the results of the practices of grape farmers and middlemen.

### 3.1. Descriptive Analysis of Grape Farmers’ and Middlemen’s Practices as Food Loss Drivers

The self-report method in socioeconomic research is vulnerable to social desirability bias, which can impact the validity and reliability of the results. To overcome (over- or underestimations), a mixed method of qualitative and quantitative methods, along with real observations, was used during data collection to ensure accuracy and avoid bias.

Socioeconomic characteristics, including age, education, gender, and cultivated area, for the selected respondents are presented in Table A2. These demographics may influence the respondents’ actions and practices, as young people with higher education can adopt best practices more quickly than older individuals. Likewise, the number of years of experience also plays a crucial role, as highly experienced farmers can determine the appropriate input quantities and types, irrigation times and water quantities, and the optimum stage of maturity for harvesting. For asset ownership, farmers and middlemen who own assets such as cars, tractors, and tricycles reported a lower loss percentage due to their ability to transport their production to or from the market, especially for small-scale farmers. At the same time, the organization’s membership proved its importance in disseminating new agricultural operations and marketing information through training, facilitating the joining of the contract farming program, and providing access to microfinance, which must be considered during the design and implementation of intervention reduction strategies.

Postharvest operations have been divided between farmers and middlemen. The farmers mainly do the upstream supply chain operations. Still, middlemen are responsible for collecting and redistributing agro-food products. Both practices determine the cumulative share of lost and wasted food downstream of the supply chain [33]. The respondents reported that during harvesting, pre-grading, and sorting, they are excluding unacceptable fruits (shapes, sizes, and colours) at the farm gate, which is consistent with the recent findings reported that the final consumers will not accept these fruits due to their shape or size and should be removed from the beginning of supply chain [10]. Furthermore, around half of the farmers (45%) used drip irrigation. They harvested the fully ripening fruits (79%) in the early morning (87%), where the drip irrigation could increase the fruit’s solidity and subsequently decrease the share of lost food across FSC. Additionally, around 72.5% of them don’t spray chemicals to accelerate fruit maturity in normal cases (although they sometimes use these chemicals to achieve a higher price, especially at the beginning of the harvest season). An overview of the main selected practices (factors) that determine the share of grape loss is presented in Table 1.

Recent studies revealed that primary inspection and sorting play a significant role in reducing losses downstream FSC [34]. Likewise, inappropriate packing boxes, such as palm crates (common for Egyptian farmers), increase the percentage of injured fruits during handling, packing, and transporting [8]. The quality indicators for grading were considered by around 67.5% of the farmers’ respondents, who were mainly based on the colour and physical blemishes of the product. Still, most of the middlemen were grading not only based on the physical blemishes but also considering (solidity, size, and weight). Furthermore, farmers and intermediaries reported that they store their production in the market (sometimes without cold storage services) before selling it for one or more days under normal temperature conditions, which increases the rejection of fruits due to color changes, wilting, insect infestation, rotting, and weight loss. Although open trucks are a common transportation mode for agricultural products in Egypt, most of the respondents reported that they don’t cover the products during transportation from the farm to the wholesale market or retailers, which increases the losses and decreases the product quality, especially, during summer and for the long-distance transportation (sometimes more than 12 h).

Most respondents reported being dissatisfied with their current marketing channel, and the primary constraint to targeting the preferred channel was a lack of market information. Access, affordability, and availability of facilitation to target the preferred marketing channel significantly impact the loss, as farmers reported an urgent need for market information and microfinance, while small intermediaries require microfinance. Most farmers either leave the injured fruits in the field or use them to feed their animals. Whereas 50% leave the injured fruit in the field or send it to the garbage, and only 33% use it for animal feeding. This appears clearly during the season or when the market price is low, the farmers prefer to leave the yield in the field or feed the animals with the unacceptable fruits [35]. However, the intermediaries mainly send them to the garbage (especially in urban and semi-urban regions), which causes multiple health and environmental impacts. This highlights the importance of minimizing losses in the upstream supply chain to reduce the share of wasted food in subsequent nodes or even at the consumer level.

### 3.2. Fractional Regression Models (FRM) Compared with OLS Results for Grapes (Farmers and Middlemen)

Improper food supply chain practices increase the amount of damaged fruit and consequently the PHL ratio at various stages (e.g., over-maturity, improper handling, overloading boxes or trucks, and improper storage) [36]. Furthermore, infrastructure availability and agro-food processing facilities are crucial in enhancing the marketing systems to decrease the PHL [37]. Hence, controlling these factors along with FSC has a tremendous and immediate impact on reducing the percentage of PHL, which could be a way to enhance the welfare of small stakeholders (farmers and intermediaries) in developing countries.

#### 3.2.1. FRM Compared with Ordinary Least Squares (OLS) Results for Grape Farmers

Identifying the optimal harvest practices to minimize losses is crucial in designing an intervention strategy. In Table 2, the results of the FRM and OLS show that the education of grape farmers positively reduces the PHL, with a 0.3% reduction in the share of lost food for FRM and a 4.9% reduction for OLS, on average, for educated farmers compared to illiterate farmers. Our current findings support the previous study that revealed the significant role of agricultural education in reducing PHL in many developing countries, such as the substantial role in the global initiative (Postharvest Education Foundation). In the past two decades, this initiative has highlighted the requirement for continuous training, policy support, and accessible technologies to sustain the impact of the PHL reduction interventions [38].

Moreover, non-use of chemicals has a strong positive effect on decreasing the PHL. It leads to an average decrease in the share of losses by 0.19% and 24% for FRM and OLS, in addition to reducing their negative health and environmental impacts. This highlights the essential role of agricultural extension services in enhancing the knowledge and skills of small farmers by training them on the best agricultural practices, including pre- and post-harvest operations [39]. In addition to the role of technical agricultural education, which significantly reduces pesticide application among vegetable farmers in China [40].

Furthermore, the irrigation system used during the production is one of the most essential factors in determining the share of PHL, where the production under drip irrigation could significantly decrease the share of lost food by 0.9% and 13.9% on average for FRM and OLS on average compared with the conventional (flood) irrigation. This is due to the low moisture and water content of grape fruits produced under drip irrigation, which deters pathogens, increases their solidity, and extends their shelf-life, subsequently decreasing losses. This agrees with a recent study that revealed that the total production under drip irrigation is higher in quantity (45%) and quality than the conventional or flood ones [41].

The results revealed that increasing the total cultivated area for the grape farmer significantly decrease the share of PHL at the grape farm level by 0.4% and 6% on average for FRM and OLS, highlighting that the increasing the cultivated land is associated with a decrease in PHL where the big farmers can benefit from (large-scale production) by lowering per-unit production and transportation costs, especially for long distances to the market. This is in line with the economic theory, where land fragmentation decreases economic efficiency and could force small farmers to delay harvesting their production to increase the quantity and reduce per-unit transportation costs [42]. This appears clearly in developing countries, but the collective marketing and cooperatives could open opportunities for small-scale grape producers to export their production or to attract local traders who secure agricultural micro-finance for them and then purchase their harvest [43]. At the same time, the large farms help farmers utilize advanced production technologies and improved varieties with a long shelf life, subsequently decreasing the share of losses.

The results of FRM revealed that harvesting early in the morning could decrease the losses by 0.7% and 8.7% on average for FRM and OLS, respectively, and it is statistically significant at 1%. Meanwhile, delaying harvest after the optimal maturity increases the share of PHL by 1.5% and 21.6% on average for FRM and OLS, respectively, compared to the optimal harvesting. Our results support the FAO findings, which revealed that the delays in harvesting due to climate, peak labor demand, and competition of Egypt’s exporters can significantly impact export volumes, diverting production to local markets and increasing the losses [2]. This highlights the role of governmental support in finding new strategies for upgrading the value chain of small-scale table grape actors to access the export market. Furthermore, the grape farmers reported that they sometimes store the harvested fruits at the farm, which increases the share of losses by 0.9% and 11.7% on average for FRM and OLS, respectively, compared to farmers who sell their production directly. In the case of storing products, previous studies have highlighted the role of using edible coatings, such as gum Arabic, in delaying ripening and maintaining fruit quality during storage [44]. For packing materials, the results revealed that the losses increase by around 0.1% and 13.7% on average for FRM and OLS, respectively, when using the crates of palm leaf compared to the carton and plastic boxes, highlighting the importance of using appropriate packing materials for slowing respiration and keeping the fruits from mechanical damage. However, there is a significant correlation between using plastic crates and lower food contamination [5]. In Egypt, the familiarity with using plastic crates is low compared to that of traditional palm crates for most small fruit and vegetable farmers. Furthermore, the packinghouse companies or stations collect grapes from the farms and then repack them according to the destination market (local supermarkets, hotels, exports). They provide these services to exporters or traders. Sometimes, they buy from grape farmers and resell the packed product to their clients, which can result in more PHL due to the repeated loading and reloading of the product [2].

The interaction between the different farmers’ practices significantly reduced the PHL. For example, combining drip irrigation and non-spraying chemicals has a negative and significant coefficient, which could decrease the PHL by an additional 1.5% and 19.8% on average for FRM and OLS, respectively. Likewise, combining the optimal harvesting time with drip irrigation results in a further reduction of 1.5% and 19.2% on average for FRM and OLS, respectively. Furthermore, using the appropriate packing and harvesting early in the morning could reduce the PHL by an additional 0.3% and 3.8% on average for FRM and OLS, respectively. Additionally, combining optimal harvesting maturity with early morning harvesting results in an average reduction of 0.6% and 8.8% for FRM and OLS, respectively. This highlights the importance of using holistic and integrated interventions to reduce the percentage of PHL, considering the cost and availability for the small farmers and stakeholders [3].

To compare the results of the FRM and OLS models, we observed that most variables align in the same direction (positive or negative) across the two models. However, significance levels vary due to methodological differences, as the Probit model assumes non-linearity and the OLS model assumes linearity. Examining the model fit, the FRM has a low pseudo R^2^ (0.0077) but a significant Wald chi^2^ (*p* = 0.0000), indicating that the model explains limited variance despite its overall significance. In contrast, the OLS model with interactions has a higher R-squared value and significant F-statistics, which suggests that linear relationships dominate the data. Where the OLS model explains ~22.1% of the log (PHL) variance, highlighting its effectiveness and suggesting better explanatory power. We expected this value to be lower for the farmers than the middlemen due to the nature of the included variables (postharvest operations done mainly by the middlemen rather than farmers), so we expect the middlemen to have a higher explanation of variance in the dependent variable (R-squared).

However, covering the products during transportation from the farm to the wholesale or local market was statistically insignificant. However, it remains one of the primary factors contributing to the increase in the PHL. However, the farm price is also statistically insignificant. Still, its fluctuations from one season to another and during the same year contribute directly and indirectly to increasing the PHL by delaying the harvest time or spraying some chemicals (sometimes) to speed up the maturity, negatively affecting product quality and increasing losses. When the farm gate price is high, the farmers try to avoid postharvest losses and keep their production as fresh as possible by covering the product or following the appropriate operations during harvesting and handling. That highlights the role of contract farming to reduce the price risk and achieve benefits for farmers and consumers; it can create linkages between them through adopting the short supply chain and using E-commerce [45].

The village’s location near the main cities plays a significant role in allowing farmers to receive training through international projects, which increases their knowledge of best practices for farm operations and post-harvest handling. This is consistent with the findings that revealed the significant role of agricultural training and extension in reducing the PHL share [39,46]. Meanwhile, increasing farmers’ experience could decrease the share of losses, especially for fruits, where farmers are familiar with water requirements, pest control, postharvest operations, and other related factors. Furthermore, we observed that new farms in the reclaimed areas produce lower yields than those in the old lands for the same varieties, possibly due to differences in soil fertility and the farmers’ experience.

#### 3.2.2. FRM Compared with OLS Results for Grape Middlemen

The main factors influencing the percentage of PHL for the grape’s middlemen are presented in Table 3. These factors include methods of storage and transportation, whether in equipped vehicles or not, as well as long-distance transportation, which affects product spoilage, as presented below.

The most essential factor is the market type (formal and informal), where the rate of losses differs according to the product’s customer and the location of the middlemen in the FSC. Trading in formal/structured markets reduces the share of lost food by ~0.7% and 13.88% on average for FRM and OLS, respectively, compared to the informal ones. This highlights the importance of a short supply chain to decrease losses and maximize marketing efficiency, which has been confirmed in the literature [47]. Regarding the gender impacts, the results showed that the PHL for male traders is ~1.8% higher than for females for FRM and 10% higher for OLS, respectively. Female traders mostly pre-sort and grade the products before reselling, compared to males. This aligns with the recent findings confirmed that female traders are more likely to follow the best handling and hygiene practices than male traders in Nigeria, contributing significantly to food safety and reducing losses [5]. Another interesting point is that we observed that most retailers and hawkers are females, and they are more likely to use plastic crates and sort products due to their dealing with small volumes, which reduces losses. Furthermore, proper storage significantly reduced losses, as evidenced by a negative and statistically significant coefficient, which is more pronounced in formal wholesale markets and supermarkets than in informal ones. Proper storage infrastructure (cold storage) is essential for reducing the PHL for perishable products like horticultural products, especially during summer [48,49].

The results revealed that the proper storage facilities could reduce the PHL by ~3.4% and 65.9% for FRM and OLS, respectively. A recent study from Nigeria showed that higher temperatures encourage microbial growth, and the number of horticultural products infested with contaminants doubled during the dry season [5]. Current results highlight significant heterogeneity of PHL in developing countries, where most losses occur between the farm gate and the consumer due to the heterogeneity of access to cold storage facilities across the FSC [50]. We observed during the market visits that the consumers (while searching and selecting the fruits) are more likely to touch and move the products, especially when they are unpackaged. This could increase food contamination and losses, especially for sensitive products like grapes, highlighting the importance of Modified Atmosphere Packaging (MAP) for such products [51]. Furthermore, the transportation mode (own or hired transportation) plays a substantial role in determining the share of PHL. The results revealed that the traders who use their own transportation can reduce the PHL by ~0.3% and 7.9% for FRM and OLS, respectively, compared with those who hire transportation. These findings suggest that the cooperatives could contribute to providing cold transportation services at subsidized fees to support small traders and farmers, thereby reducing their losses.

The longer distance to market results in a higher PHL, where the PHL increases 0.008% and 0.15% on average for an increase in the distance for the FRM and OLS, respectively. The increased transportation risks or delays lead to higher quantity and quality losses caused by the growth of fungi and bacteria. Due to the use of open trucks and the long distance between the production site and the wholesale or retail market, most farmers reported that they didn’t cover the products during their journey to the markets. The sunshine caused massive damage to the grapes and increased the PHL, which is in line with the findings of a recent study [52]. Likewise, a recent study revealed that the main determinants of food losses in developing countries are the lack of infrastructure, inadequate marketing systems, and improper handling practices, highlighting the role of public-private investment [53]. The long distance and unavailability of cold transportation increase the possibility of losses, so the accessibility of refrigeration and a steady cold chain are necessary to prevent both qualitative and quantitative losses in fresh products, such as fruits and vegetables [54]. The efficiency of the cold chain depends on adjusting product temperature at each step and managing worker practices, ensuring a clean cold chain for a sustainable PHL reduction strategy [49]. Refrigerated vehicles are expensive for small farmers because they often cannot access microfinance, but they can access these services through cooperatives. In Egypt, exporters have identified the shortage of packaging, pre-cooling, and cold storage facilities as a key factor restricting trade. The shortage of cold-chain storage services on farms and in the market represents the primary challenge for small farmers and intermediaries across the horticultural supply chain in developing countries [53].

The percentage of stored products for the next day outside the cold storage causes a decrease in the quantity & quality, and increases PHL [55]. The production source for middlemen plays an essential role in the PHL percentage, where local traders sometimes spend a long time collecting produce from small farmers, which negatively affects product quality and increases food loss in subsequent stages. This is consistent with [5]. The total quantity that the trader deals with (buying and selling) is one of the essential factors that can lead to an increase in the PHL, and sometimes result in more losses of net profit for the different middlemen due to quality deterioration. Meanwhile, the losses increase as the time of loading and unloading the products during transportation and handling due to the lack of agricultural marketing facilities and information exchange, which are crucial in moving agricultural products from the farm gate to the final consumer; this will benefit all actors across the FSC [21,56,57].

The interaction between the different middlemen’s practices significantly reduced the PHL. For example, combining the market type and proper storage facilities could decrease the PHL by an additional 0.5% and 10.6% for the FRM and OLS, respectively. Likewise, combining access to adequate storage facilities with the use of the middleman’s transportation results in an additional reduction of 0.7% and 15% on average for the FRM and OLS, respectively. This highlights the importance of using holistic and integrated interventions to reduce the percentage of PHL, considering the cost and availability for the small farmers and stakeholders.

Examining the model fit, the FRM has a low pseudo-R^2^ (0.0089) but a significant Wald chi^2^ (*p* = 0.0000), indicating that the model explains limited variance despite overall significance. In contrast, the OLS model with interactions has a higher R-squared value and significant F-statistics, which suggests that linear relationships dominate the data. Where the OLS model explains ~52.6% of the log (PHL) variance, highlighting its effectiveness and suggesting better explanatory power. R-squared for the middlemen is higher than that of the farmers (higher explanation of variance in the dependent variable (%PHL), because of the nature of the included variables (postharvest operations done mainly by the middlemen rather than farmers). Although some factors, such as education, access to finance, and experience, were statistically insignificant in this model, they still explain a significant portion of the variance between stakeholders in the PHL. This highlights the need for future studies to better understand the interaction between these factors as drivers of food losses at the middlemen level.

**Figure 2:**
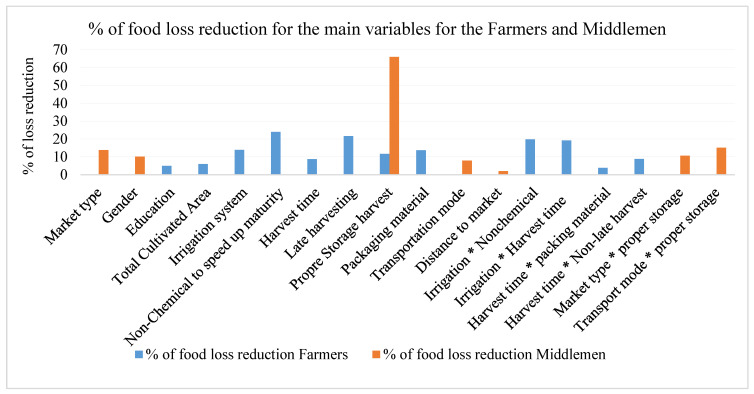
Percentage of loss reduction according to the results of the current study for the Farmers and Middlemen.

Overall, this study found a statistically significant correlation between improper pre- and post-harvest practices and PHL between Egyptian grape farmers and middlemen. The current results confirm the application of the fundamentals of the theory of rational choice to farmers and middlemen for investing in PHL reduction. This highlights the manuscript’s theoretical contribution through the empirical estimations in this field. However, the adoption rate of best recommended postharvest practices remains low among farmers and middlemen. Efforts to disseminate and encourage stakeholders to adopt these best-practice recommendations can significantly reduce the share of PHL in grapes and other fresh fruit and food products. We concluded the percentage of the main significant variables of the current results in Figure 2.

## 4. Conclusions

Poor postharvest practices, stemming from insufficient resources and knowledge, significantly contribute to losses throughout the food supply chain. Improving these practices is crucial for reducing FLW and enhancing the well-being of farmers and other stakeholders in developing nations. This study aims to investigate the impact of pre- and post-harvest practices on reducing food loss among Egyptian grape farmers and middlemen. Using a combination of the Fractional Regression Model and the Ordinary Least Squares model, the study found strong correlations between socioeconomic characteristics and stakeholders’ practices at various stages of the food supply chain. The study also found that drip irrigation, delayed harvesting, and the use of appropriate packing materials can reduce losses at the farmer level. The study also found that farmers’ education and cultivated area significantly influence PHL, with increased education and cultivated area leading to a decrease in PHL. Furthermore, the study emphasizes the importance of integrated interventions to minimize pesticide use in grape production, highlighting the need for further research to explore the economic feasibility of these combined approaches for small farmers.

For the agricultural markets, the study reveals that formal agricultural markets reduce post-harvest loss (PHL) by 13.88% compared to informal markets, underscoring the importance of efficient supply chains. Gender disparities and a lack of cold storage contribute to losses. Transportation mode and distance to markets also impact PHL. The study emphasizes the need for accessible refrigeration and a robust cold chain, including temperature control and hygienic practices at each stage. While refrigerated vehicles are expensive, microfinance through cooperatives could offer a solution. Inadequate packaging, pre-cooling, and cold storage facilities pose significant constraints, particularly for small-scale farmers and intermediaries. Finally, these findings underscore the importance of integrating market-type improvements with suitable storage facilities and transportation to reduce postharvest loss (PHL) by 10.6% and 15%, respectively. It emphasizes the need for holistic strategies that consider both cost and accessibility for all stakeholders. Proper storage adoption yields the highest marginal returns for quality, while subsidies for technologies like improved packing can mitigate losses. Agricultural cooperatives offer cost-effective cold storage and transportation services, while intensive training programs for farmers and intermediaries are also crucial. These findings can inform policymakers and agribusiness managers in designing effective, cost-efficient strategies to reduce PHL, boost smallholder profits, and foster sustainable food systems.

## 5. Limitations and Future Research Direction

Improved post-harvest handling, including harvesting, sorting, and the use of cold chain technologies, significantly reduces food losses in developing countries. Still, due to the lack of data, measuring the percentage of PHL and its impacts at different FSC nodes is challenging, highlighting the need for further investigations using primary data in the future to explore the leading causes according to different crops in various regions. Although we attempted to minimize the risk of social desirability bias in the self-reported methodology and due to heterogeneity among stakeholders, regions, crops, and the varying importance of different PHL causes, the current results may be limited in their generalizability to other crops in other areas. Investment in agrofood logistics, facilitated by public-private partnerships, is crucial for extending shelf life and enhancing the profitability of smallholder farmers. However, public-private coordination faces numerous challenges due to the weak institutional structures in developing countries, highlighting the need to investigate the impacts of public policy on the sustainability of agrofood systems. Public agricultural extension is vital in disseminating best practices among farmers. Still, the number of agrarian facilitators decreases over the years in most developing countries (which could affect the estimation accuracy and the applicability of the suggested reduction interventions).

Further investigations are needed to assess the socioeconomic and environmental implications of integrating various farming practices with middlemen, thereby achieving the highest marginal returns for quality, minimizing losses, and enhancing supply chain efficiency. This framework could provide policymakers and agribusiness managers with more reliable, applicable, and economical insights for designing effective interventions. Future research should prioritize the role of the contract farming system in reducing post-harvest losses and securing additional resources for small farmers, which could help minimize the severity of market fluctuations. Innovation plays a significant role in securing low-cost logistics services for small-scale stakeholders, as well as proper handling and temperature control, which can reduce losses. This requires further investigation to ensure the applicability and economic viability of the proposed solutions.

## Figures and Tables

**Table 1:** Descriptive analysis of the pre- and post-harvest practices for grapes (farmers’ and middlemen).

Stage			Farmers	Middlemen
	Items	Description	% Frequencies N = 200	% Frequencies N = 120
**Farm** **operations**	**Irrigation system**	Drip	45	-
		Conventional	55	-
	**Spray chemicals to speed up the maturity**	Yes	27.5	-
		No	72.5	-
**Harvesting**	**Harvesting time of the day**	Early in the morning	87	-
		Any time of the day	13	-
	**Stage of maturity**	Fully ripe	79	-
		Partially ripe	21	-
	**Delay harvesting (after optimum maturity)**	Yes	56.5	-
		No	43.5	-
	**The reason**	To catch a high price	50.0	-
		To avoid the low-price	40.5	-
		None	9.5	-
	**Method of harvesting**	Scissors/knife	49.5	-
		Hand	50.5	-
		Machinery	-	-
**Sorting and grading**	**Grade/pre-sort your produce immediately**	Yes	58.5	70
		No	41.5	30
	**Quality indicators for grading**	Color	29.5	2.5
		Size	3.0	-
		Weight	19.0	-
		Physical blemishes	38.0	52.3
		All the above	10.5	39.2
**Storage**	**Access to proper storage facilities**	Yes	50.5	88.3
		No	49.5	11.7
	**Storage problems with your produce?**	Colour changing	18.5	-
		Wilting and shrinking	6.0	-
		Weight losses	6.0	2.5
		Insects and rotting infestations	7.0	45
		All the above	62.5	52
	**Storage place**	At farm	19	-
		At home	3.6	-
		In the market	77.4	100
	**Number of storage days, **farmers **(N = 128),** For middlemen **(N = 107)**	One Day	81.5	38.3
		Two days or more	18.5	61.7
**Packing**	**Packaging material**	Wood Box	-	-
		Crates of palm leaf	27.0	72
		Carton Box	30.0	-
		Plastic Box (improved packing)	43.0	28
**Transporting**	**Product coverage during transportation?**	Yes	28.5	-
		No	31.5	52.5
		Sometimes	40.0	47.5
	**Distance to the nearest market** **(**Km**)**	50–100	-	80.8
		101–150	1.5	4.2
		151–200	21.0	11.7
		<200	77.5	3.3
	**Transportation time**	Early in the morning	56.5	47
		Any time of the day	43.5	53
	**Transportation mode**	Own transportation	12.3	23
		Hired transportation	87.7	77
**Marketing**	**Type of market**	Formal market	23	56
		Informal market	77	44
**Dealing with losses during the different stages?**		Donate to charity	11.5	-
		Feed animals	33.0	31.7
		Compost	5.0	-
		Landfill (left in the field)	22.0	-
		Garbage	28.5	68.3
**Do you need facilitation to target the preferred marketing channel?**		Cold-Transportion	15.5	1.8
		Equipped storage places	19	14.5
		Microfinance	14.0	50.9
		Market information	45.5	13.6
		None	6	19.2
**Are you satisfied with your current main marketing channel?**		Yes	16	47.5
		No	84	52.5
**What are the constraints to target the preferred marketing channel?**		Lack of marketing information	70.1	-
		High cost	19.2	-
		Needs big quantity	10.7	-

**Table 2:** Fractional probit regression results (grapes farmers).

Farmers	Fractional Probit Regression			OLS Estimations	
PHL	Coef.	Std. Err.	Marginal Effect (dy/dx)	Coef.	Std. Err.
Cons.	−1.9172	0.30926 ***	-	−3.07161 ***	0.51874
Age	−0.000896	0.0018351	−0.00308	−0.00258	0.00431
Education	−0.02017 *	0.011534	−0.00308	−0.04894	0.02938
Experience	−0.0006893	0.003843	−0.0001	−0.00040	0.00938
Total Cultivated area	−0.02872 **	0.011849	−0.00438	−0.06116 **	0.02971
Grapes Cultivated	−0.008235	0.015477	−0.00125	−0.013729	0.04151
Information Source	−0.0004830	0.008711	−0.00073	−0.01184	0.02157
Cooperative membership	−0.0374406	0.03239	−0.00571	−0.08225	0.07927
Access to microfinance	−0.0235572	0.03289	−0.00359	−0.08205	0.07880
Contract farming	−0.0385765	0.03430	−0.00588	−0.07118	0.08804
Irrigation system	−0.05994 *	0.03458	−0.00915	−0.13909 **	0.07874
Non-Chemical to speed up maturity	−0.12198 ***	0.03352	−0.01862	−0.24078 ***	0.08370
Harvest Method	−0.01706	0.03375	−0.0026	−0.00731	0.07672
Harvest time	−0.04546 **	0.02052	−0.00694	−0.08752 **	0.04600
Late harvesting	0.10239 ***	0.03033	0.01563	0.21671 ***	0.07474
Stage maturity	−0.04359	0.041401	−0.0066	−0.07258	0.09656
Presort and Grade immediately	−0.02042	0.03386	−0.00311	−0.04036	0.07695
Propre Storage harvest	−0.06058 *	0.031385	−0.00924	−0.1173	0.07637
Packaging material	−0.06801 ***	0.018199	−0.01038	−0.13785 ***	0.04685
Cover Product	−0.02572	0.02074	−0.00392	−0.05611	0.04795
Distance to market	0.0004	0.000262	0.0006	0.00003	0.00061
Farm price	−0.00851	0.038608	−0.00130	−0.00355	0.09433
**Interaction effect**					
Irrigation * Nonchemical	−0.0978 ***	0.01770	−0.01498	−0.19834 ***	0.04349
Irrigation * Harvest time	−0.09933 ***	0.02145	−0.01521	−0.19248 ***	0.05509
Harvest time * packing material	−0.02140 **	0.01087	−0.00327	−0.03803 **	0.02328
Harvest time * Non-late harvest	−0.04080 ***	0.01650	−0.00625	−0.08877 ***	0.04167
	N = 200 Wald chi2 (21) = 106.69 Prob > chi2 = 0.0000 Pseudo R^2^ = 0.0077			F(21, 178) = 2.39 Prob > F = 0.0011 R-squared = 0.2201 Adj R-squared = 0.1281	

*, **, and *** are refer that *p* < 0.1, <0.05, and <0.01.

**Table 3:** Fractional probit regression results (grapes middlemen).

Middlemen	Fractional Probit Regression			OLS Estimations	
	Coef.	Std. Err.	Marginal Effect (dy/dx)	Coef.	Std. Err.
Cons.	−1.3841 ***	0.19093		−2.35058 ***	0.44873
Market type	−0.06916 ***	0.01320	−0.00775	−0.13881 ***	0.0363
Gender	0.05423 **	0.02522	0.00620	0.10136 *	0.06671
Age	−0.00059	0.00117	−0.000067	−0.00024	0.00295
Education	−0.01619	0.01349	−0.00183	−0.03823	0.03126
Experience	−0.02731	0.02203	−0.00310	−0.06235	0.06735
cooperative	−0.06501	0.04692	−0.00738	−0.14608	0.09549
Access to microfinance	−0.03843	0.04139	−0.00436	−0.07905	0.08440
Information source	−0.01274	0.021062	−0.001447	−0.0313615	0.04021
Proper Store harvest	−0.30398 ***	0.04929	−0.03468	−0.6593 ***	0.10466
Transportation time	0.00702	0.01234	0.00079	0.00547	0.04684
Pre-sort and -grade immediately	−0.01341	0.04148	−0.00152	−0.02186	0.081292
Transportation mode	−0.03435 ***	0.01200	−0.00382	−0.07932 **	0.03548
Packaging material	−0.067853	0.03223	−0.00680	−0.00680	0.03209
Cover product	−0.01486	0.02400	−0.00168	−0.03810	0.05689
Distance to market	0.00072 ***	0.00027	0.00008	0.00158 ***	0.00046
**Interaction effect**					
Market type * proper storage	−0.05233 ***	0.01122	−0.00596	−0.10655 ***	0.030337
Transport mode * proper storage	−0.06623 ***	0.01312	−0.00754	−0.15159 ***	0.03641
	N = 120 Wald chi^2^ (15) = 166.88 Prob > chi^2^ = 0.0000 Pseudo R^2^ = 0.0089			F (15, 104) = 7.69 Prob > F = 0.000 R-squared = 0.5258 Adj R-squared = 0.4574	

*, **, and *** are refer that *p* < 0.1, <0.05, and <0.01.

## Data Availability

The original contributions presented in the study are included in the article, further inquiries can be directed to the corresponding authors.

## Abbreviations

The following abbreviations are used in this manuscript:

PHL	Postharvest Losses
FSC	Food Supply Chain
FLW	Food Losses and Waste
RCT	Rational Choice Theory
OLS	Ordinary Least Squares
FRM	Fractional Regression Model

## Appendix A

**Table A1:** Summarydescription of the main PHL explanatory variables.

Variable	Description
**Stakeholders’** **Practices and Technological Factors**	
**Irrigation system (IRR)**	(1) Drip (0) Conventional
**Harvesting Time during the day (HT)**	Early in the morning
	Any time across the day
**Quality indicators for grading (QI)**	Color
	Size
	Weight
	Physical blemishes
	All the above
**Acess for proper storage facilities (SF)**	Yes No
**Storage problems of your produce? (SP)**	color changing
	wilting and shrinking
	weight losses
	insects and rotting infestation
	All the above
**Facilitation do you need to target the preferred marketing channel? (FMC)**	Cold Transportion
	Equipped Storage places
	Microfinance
	Market information
	None
**Dealing with losses during the different stages? (DPHL)**	Donate for charity
	Feed animals
	Compost
	Landfill (left at the field)
	Garbage
**Delay harvesting after optimal maturing? (DH)**	Yes No
**The reason (RED)**	To get a high price
	To avoid the low-price
	None
**Cover Product during transportation? (CP)**	Yes No Sometimes
**Harvest Method**	(a) By knife cutting (b) by hand twisting (c) Machinery Harvesting
**What stage of maturity of the fruit do you harvest?**	(a) Fully matured (b) Partially matured
**Grade/pre-sort your produce immediately**	Yes No
**Number of storage days**	Days
**Spray chemicals to accelerate the maturity**	Yes No
**Packaging material**	1. Wood Packaging Box 2. Crates of palm leaf 3. Carton Packaging Box 4. Plastic Packaging Box (improved packing)
**Distance to market**	Kilometers
**Type of the market**	Formal market
	Informal market
**Transportation time**	Early in the morning
	Any time of the day
**Transportation mode**	Own transportation
	Hired transportation
**Stakeholders’ Socioeconomic Characteristics**	
**Gender**	Male Female
**Age**	Years
**Education level**	1 = university or college or equivalent 2 = secondary school (technical training) 3 = Primary/preparatory school 4 = Only read and write 5 = Illiteracy
**Marital status**	1. Married 2. Single 3. Divorced 4. Widower
**Family members Number**	Number
**Experience Years**	Years
**Total Cultivated Area**	Feddan (4200 m^2^)
**Cultivated area with study crop**	Feddan
**Source of information**	1. Friends 2. Family members 3. Neighbors 4. Market agencies 5. Agricultural cooperation and extension 6. Companies and Inputs distributors
**Member of an organization or cooperative**	Yes No
**Borrowed a loan (Access to finance)**	Yes No
**Contract to sell your produce**	Yes No
**Are postharvest losses a problem for you?**	Yes No
**Total_Production**	Kg/feddan
**Farm gate price per Kg**	LE/kg
**PHL_percent**	**% (Dependent variable)**

**Table A2:** Sample description and descriptive statistics for the investigated respondents (grape farmers and Middlemen).

Stakeholders		Farmers N = 200		Middlemen N = 120									
				Village Trader N = 30		Wholesaler N = 25		Fruit Grocery N = 30		Supermarket N = 10		Hawker N = 25	
Items	Description	F	%	F	%	F	%	F	%	F	%	F	%
**Gender**	Male	196	98	27	90	25	100	6	12	14	70	31	62
	Female	4	2	3	10	-	-	44	88	6	30	19	38
**Age**	20 to <30 years old	35	17.5	3	10	-		8	16	4	20	6	12
	30 to <40	56	28	10	33.3	6	12	4	8	6	30	4	8
	40 to <50	60	30	14	46.7	24	48	22	44	5	25	26	52
	50 to <60	39	19.5	3	10	16	32	12	24	5	25	8	16
	>60	10	5	-	-	4	8	4	8	-	-	6	12
**Education level**	University	16	8	4	13.3	5	10	-	-	8	40	-	-
	Secondary school	28	14	4	13.3	9	18	5	10	11	55	4	8
	Primary/preparatory school	38	19	3	10	7	14	5	10	1	5	7	14
	Adult education/Read and write	61	30.5	11	36.7	19	38	30	60	-	-	9	18
	Illiterate	57	28.5	8	26.6	10	20	10	20	-	-	30	60
**Marital status**	Married	184	92	25	83.3	47	94	30	60	15	75		90
	Single	14	7	2	6.7	-	-	2	4	3	15	1	2
	Divorced/Widower	2	1	3	10	3	6	18	36	2	10	445	8
**Number of Family members**	2–4	36	18	9	30	15	60	12	41	7	70	7	28
	5–7	118	59	18	60	8	32	15	50	3	30	15	60
	>7	46	23	3	10	2	8	3	9	-	-	3	12
**Experience Years**	<5 years	14	7	-	-	-	-	5	16.7	3	30	7	23.3
	5 to <10	68	34	13	43.3	5	16.7	12	40	2	20	18	60
	10 to <15	76	38	12	40	7	23.3	10	33.3	4	40	5	16.7
	>15	42	21	5	16.7	18	60	3	10	1	10	-	-
**Source of Information**	Friends	60	30	7	23.3	15	60	10	33.3	-	-	13	52
	Family members	6	3	-	-	-	-	-	-	-	-	-	-
	Neighbors	14	7	10	33.3	5	20	-	-	-	-	7	28
	Market agencies	60	30	10	33.3	4	16	17	56.7	6	60	5	20
	Cooperation and agro-extension	13	6.5	3	10	1	4	-	-	-	-	-	-
	Companies and Inputs’ distributors	48	24	-	-	-	-	3	10	4	40	-	-
**Total Land Ownership**	<1 *Feddan*	70	35	-	-	-	-	-	-	-	-	-	-
	1–3 *Feddan*	96	48	-	-	-	-	-	-	-	-	-	-
	More than *3 Feddan*	34	17	-	-	-	-	-	-	-	-	-	-
**Grapes Cultivated Area**	<3 *Feddan*	116	58	-	-	-	-	-	-	-	-	-	-
	3–5 *Feddan*	52	26	-	-	-	-	-	-	-	-	-	-
	More than 5 *Feddan*	32	16	-	-	-	-	-	-	-	-	-	-
**Member of an organization or cooperative**	Yes	98	46	5	16.7	15	60	3	10	8	80	0	0
	No	112	54	25	83.3	10	40	27	90	2	20	25	100
**Borrowed a loan (Access to finance)**	Yes	118	59	8	26.6	20	80	4	13.3	6	60	2	8
	No	82	41	22	73.3	5	20	26	86.7	4	40	23	92
**Contract to sell your produce**	Yes	24	12	10	33.3	18	72	-	-	-	-	-	-
	No	176	88	20	66.7	7	28	-	-	-	-	-	-
**Are post-harvest losses a problem for you?**	Yes	174	87	30	100	19	76	10	33.3	10	100	7	28
	No	26	13	0	0	6	24	20	66.7	0	0	18	72
**Total Production**	Ton/feddan *	8.9 Ton/fed											
**Farm gate price per Kg**	LE/kg **	5.34		6.14		6.82		8.7		8.14		9.17	
**PHL_percent**	**%**	6.12%		1.65%		2.11%		2.7%		2.4%		1.9%	

* Feddan = 4200 square meters, it is the common unit of land area in Egypt. ** The average of annual exchange rate 2020. 1 $ = 15.7562 Egyptian pound. Central Bank of Egypt (CBE) https://www.cbe.org.eg/ (accessed on 30 April 2025).
